# The Turpentine Treatment of Enteric Fever

**Published:** 1876-05

**Authors:** 


The Turpentine Treatment of Enteric Fever.—Dr. A. Moffitt writes to the *Lancet:* The regiment of which I have medical charge arrived in India on the 18th of March, 1875, and between that time and the end of the year seventeen persons were attacked with enteric fever. Of this number five were treated in the ordinary way, and in them the disease ran a long and severe course, many complications arising during its progress, and finally proved fatal in two out of the five cases, which is about the usual mortality in this country. The remaining twelve patients were treated with turpentine, and in them the disease ran a short and mild course, with few complications; and all the symptoms, but especially the enteric, were so modified that no fears were entertained during its progress of an unfavorable termination, and the twelve patients, without an exception, made good recoveries.

The line of treatment pursued was as follows: As soon as the disease was diagnosed, oil of turpentine in half-drachm doses, made up with mucilage of eggs, was given four times a day, and continued throughout its course; and nourishment in the liquid state, such as beef-tea, milk, chicken-broth, eggs beaten up, etc., was given freely, and a stimulant, generally brandy, was given as the symptoms indicated, but less in the cases that occurred latterly than formerly. The turpentine treatment of enteric fever was first brought to my notice by Dr. Moffitt, of Sydney, where the disease is always prevalent. As to its *modus operandi* I would not yet hazard an opinion. Dr. Moffitt claims for it a specific action. Whatever its mode of action, if it prove as successful in the hands of others as it has done in mine and in Dr. Moffitt's practice, I have every hope it will take a place in the treatment of enteric fever similar to that taken by ipecacuanha in the treatment of dysentery, and quinine in the treatment of malarial fever. When the disease assumes a remittent character, which it will do where the patients have been subject to malarial influences, Dr. Moffitt combines it with quinine, and thinks it acts better. *Medical and Surgical Reporter.*

